# Induction of immune gene expression and inflammatory mediator release by commonly used surgical suture materials: an experimental in vitro study

**DOI:** 10.1186/s13037-017-0132-2

**Published:** 2017-05-31

**Authors:** Alistair M. Lock, Ryan Gao, Dorit Naot, Brendan Coleman, Jillian Cornish, David S. Musson

**Affiliations:** 1 Department of Medicine, University of Auckland, Private Bag 92019, Auckland, 1142 New Zealand; 2 Department of Orthopaedics, Middlemore Hospital, Private Bag 93311, Auckland, New Zealand

**Keywords:** In vitro, Immune response, Suture materials, Wound closure

## Abstract

**Background:**

Surgeons have a range of materials to choose from to complete wound closure, yet surprisingly very little is still known about the body’s immune response to the suture materials in current use. The growing literature of adverse suture material reactions provided the objective of this study, to use in vitro assays to quantify levels of inflammation produced by seven commonly used suture materials in surgical procedures.

**Methods:**

Human monocyte/macrophage THP-1 cells were exposed to suture materials for 1, 3 and 5 days. Gene expression and protein secretion of six inflammatory cytokines and two cell surface markers were assessed using qPCR and ELISA respectively, with LPS exposure providing a positive control. Furthermore, a IL-1β/IL-1RA marker ratio was assessed to determine the balance between pro-/anti-inflammatory expression.

**Results:**

The findings from our in vitro study suggest that four commonly used suture materials cause upregulation of pro-inflammatory markers indicative of an early foreign body reaction, with no balance from anti-inflammatory markers.

**Conclusions:**

As prolonged early pro-inflammation is known to produce delayed wound healing responses, the knowledge produced from this study has potential to improve informed surgical decision making and patient safety. This work has the capability to reduce suture-related adverse immune reactions, and therefore positively affect patient outcomes.

## Background

Surgeon’s have a wide range of suture materials to choose from to complete wound closure. These are mostly synthetic monofilament or polymeric sutures, which have replaced traditional natural-based sutures due to the natural sutures being associated with inflammation and hypersensitivity reactions [1–4]. Most non-absorbable sutures remain in situ for periods of at least 10 days, while absorbable options remain for many months, yet surprisingly little is known about the body’s immune response to the suture materials in current use [5, 6].

There have been many published cases where foreign body reactions to suture material have caused complications and rejection post-surgery, both in the short and long-term. Warme et al. (2004) reported the first case of a foreign body reaction to Ticron® suture, with a resultant localised granulomatous abscess requiring drainage and re-operation three-years post-surgery [7]. Moreover, five cases of sinus formation and granulomatous reaction to FiberWire® suture have been reported at sites of lower leg amputation [8]. These suture related granulomas were discovered between 5–16 months post-surgery. To date, there have also been five reported cases of suture-related pseudoinfection (SRPI) and granuloma formation following wound closure with the VICRYL® suture material, all less than ten weeks post total hip arthroplasty. These cases all required debridement and surgical revision [9, 10].

All of these cases ruled out infection with negative culture, and attributed patient outcome solely to adverse suture material reaction following histological examination of the inflammatory masses. However, the true scale of the adverse suture material reaction is likely much larger than what is available in the literature, as cases will often go unreported.

The current literature is in substantial need of immune response data for such a wide range of suture materials. Given the global push for a reduction in animal studies [11], in vitro studies offer a viable and ethical alternative for obtaining this required data. However, such studies have been few in number. One early published report measured immune response in vitro using peritoneal macrophages originally isolated from Wistar rats, and showed significantly increased tumour necrosis factor alpha (TNFα) production from cells exposed to Mersilk®, PDS II® and VICRYL® sutures [12]. In a more recent study, Musson et al. (2015) isolated primary human dendritic cells to evaluate the immune response of silk-based tendon biomaterial scaffolds in vitro, and used the suture material FibreWire® as a clinical control. This study used cytometric bead array analysis to measure concentrations of multiple pro-inflammatory cytokines in the cell conditioned media and found that concentrations of interleukin-1 beta (IL-1β), interleukin-6 (IL-6), interleukin-10 (IL-10), interleukin-12 (IL-12) and TNFα were elevated in cells exposed to FibreWire®, compared to baseline negative control [13].

Here, we use in vitro assays to quantify levels of inflammation produced by seven common suture materials; VICRYL®, MONOCRYL®, Ethilon®, Ti-Cron®, FibreWire®, Perma-Hand® and Ethibond EXCEL®. Our aim is that the information obtained from this study be provided to surgeons, to improve informed surgical decision making and patient safety.

## Methods

### Suture preparation

Seven suture materials (VICRYL®, MONOCRYL®, Ethilon®, Perma-Hand®, Ethibond EXCEL®, (Ethicon Inc.®, New Jersey, USA), Ti-Cron® (Covidien®, Dublin, Republic of Ireland) and FiberWire® (Athrex®, Florida, USA)) were sourced from our local hospital. Suture materials were obtained pre-sterilised by gamma-irradiation and packaged for clinical use. Respective sutures were cut into homogenous 5mm sections, placed into 24-well tissue culture plates, and pre-soaked in Roswell Park Memorial Institute (RPMI) cell media (Invitrogen™, ThermoFisher Scientific, Australia) for 24 hours. Sterile conditions were maintained in class II laminar flow hoods.

### THP-1 cell culture

Monocytic THP-1 cells were sourced from the American Tissue Culture Collection (THP-1, TIB-202™, ATCC®). Human monocyte/macrophage cell line THP-1 cells were cultured in 75cm^2^ tissue culture flasks with 30mL RPMI media supplemented with 10% fetal bovine serum (FBS) (HyClone Laboratories, Utah, USA), 10,000U/mL Penicillin/Streptomycin mixture (Gibco, ThermoFisher Scientific Inc., Massachusetts, USA) and 1mM Sodium Pyruvate. Cells were maintained at 37°C inside a humidified atmosphere of 5% CO_2_:95% air until the required number of cells were present. The cells were collected in 50mL falcon tubes and centrifuged at 12000rpm for two minutes. Cell pellets were then resuspended for a second time in fresh RPMI media. Finally, a haemocytometer was used to estimate cell number, and the cell suspension diluted in cell culture media to achieve a 1.5million cells/mL concentration.

1.5 million THP-1 cells (1mL cell suspension) were seeded onto each of the pre-prepared suture materials inside 24-well tissue culture plates, as described above (*n* = 4). Additional wells in the plates were allocated for negative and positive controls (*n* = 4). The negative control wells consisted of 1.5 million THP-1 cells cultured alone, whilst the positive control wells consisted of 1.5 million THP-1 cells treated with 5 ng/mL lipopolysaccharide (LPS, Sigma-Aldrich NZ Ltd). Plates were incubated at 37°C inside a humidified atmosphere of 5% CO_2_:95% air. Culture time periods were 1, 3 and 5 days post cell-seeding.

### Gene expression analysis

RNA for real-time quantitative polymerase chain reaction (qPCR) was prepared and combined from both adherent and non-adherent THP-1 cells exposed to the suture materials for 1, 3, and 5 days. Cells were lysed with β-mercaptoethanol in RLT buffer (QIAGEN Pty Ltd, VIC, Australia) and incubated at 55°C with 0.2mg/ml proteinase K (Invitrogen™, Life Technologies) for 15 minutes. 80% v/v ethanol was added and RNA extracted using the RNeasy mini kit (QIAGEN). Genomic DNA was removed from all RNA preparations with the RNase-free DNase set (QIAGEN). The quantity and purity of the RNA were measured using a Nanodrop™ Lite Spectrophotometer (Thermo-Scientific, Massachusetts, USA), with a 260/280 absorbance value of >1.8 being considered acceptable. 500ng RNA was used to make cDNA. cDNA was synthesized with Superscript III (Invitrogen™, Life Technologies) in the presence of an RNase inhibitor (RNaseOUT™, Invitrogen™, Life Technologies) and used for quantitative real-time polymerase chain reaction (qPCR), with a Quantstudio™ 12K Flex Real-Time PCR System (Applied Biosystems by Life Technologies). Primers and probe sets were purchased as fluorescently labelled TaqMan® Gene Expression Assays (Cat. # 4331182, Invitrogen™, Life Technologies). All probes used to detect target genes were labelled with FAM™ and the 18S rRNA endogenous control probe was labelled with VIC®. The ΔΔCt method was used to calculate the relative levels of expression compared to a control sample from day one [14]. All probes used span exon-exon junctions thus do not detect genomic DNA, and negative control (no cDNA) reactions were also used for each of the TaqMan® assays, without a positive signal.

Six inflammatory cytokines and two cell surface markers genes were assayed. Interleukin-1 alpha (IL-1α) (Hs00174092_m1), IL-1β (Hs00174097_m1), TNFα (Hs01113624_g1) and interleukin-8 (IL-8) (Hs00174103_m1) are pro-inflammatory cytokines produced by macrophages of the M1 (classically activated) population [15–17]. C-C chemokine receptor type 7 (CCR7) (Hs01013469_m1) is a cell surface marker found on activated M1 macrophages [17]. Transforming growth factor beta (TGFβ1) (Hs00998133_m1) and interleukin-1 receptor antagonist (IL-1RA) (Hs00893626_m1) are anti-inflammatory cytokines produced by macrophages of the M2 (alternatively activated) population [15–17]. Cluster of differentiation 163 (CD163) (Hs00174705_m1) is a cell surface marker found on M2 macrophages [17].

### Protein secretion analysis

IL-1β protein concentrations from the conditioned cell media of THP-1 cells exposed to suture materials were analysed using an R & D systems human IL-1β/IL-1F2 Duoset Enzyme-Linked Immunosorbent Assay (ELISA) kit (Pharmaco NZ Ltd.). THP-1 cell-free media was diluted out 1 in 2, and 1 in 4 per sample, using RPMI media to allow comparisons to neat cell-free media. 100μl of each neat sample and respective dilutions were used inside 96-well plates. Standard antibody was diluted in 1% bovine serum albumin in phosphate buffered saline (reagent diluent). Pure reagent diluent was used as a negative control. Plates were read on a Synergy 2 Plate Reader (Biotek®, Vermont, USA) at 450nm absorbance. Final IL-1β protein concentrations were determined in pg/ml.

### Statistical analysis

Both gene expression and protein secretion analyses were repeated twice to allow for technical variation, with three independent biological experiments performed per suture material. Data were analysed using two-way analysis of variance (ANOVA) with *post hoc* Dunnett’s test, using GraphPad Prism Software (GraphPad Software, California, USA).

## Results

### Gene expression analysis

THP-1 cells were cultured with the chosen suture materials and harvested on days 1, 3 and 5. The relative gene expression of six inflammatory cytokines and two cell surface markers were measured by qPCR. The most representative biological repeat is reported.

#### Pro-inflammatory markers

**Fig. 1:**
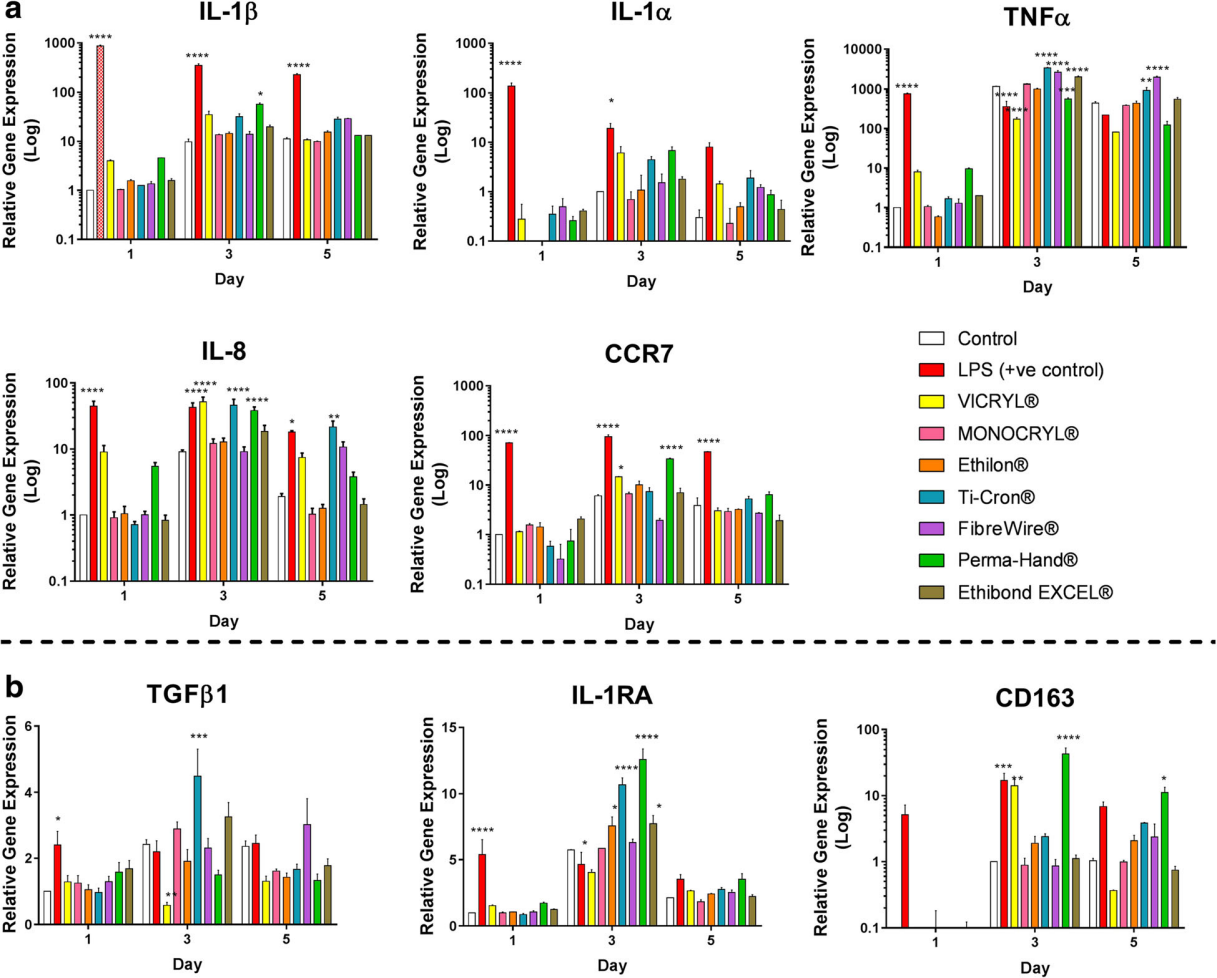
**a** Gene Expression Results. Relative gene expression of five pro-inflammatory markers (four cytokines (IL-1β, IL-1α, TNFα and IL-8) and one cell surface marker (CCR7)) from THP-1 cells in contact with a variety of suture materials were measured by RT-PCR on days 1, 3 and 5. The most representative independent biological repeat is reported ± SEM. Statistical analysis was performed using two-way ANOVA and post-hoc Dunnett’s test (*p < 0.05, **p < 0.01, ***p < 0.001), ****p < 0.0001). **b** Gene Expression Results. Relative gene expression of three anti-inflammatory markers (two cytokines (TGFβ1 and IL-1RA) and one cell surface marker (CD163)) from THP-1 cells in contact with a variety of suture materials were measured by RT-PCR on days 1, 3 and 5. The most representative independent biological repeat is reported ± SEM. Statistical analysis was performed using two-way ANOVA and post-hoc Dunnett’s test (*p < 0.05, **p < 0.01, ***p < 0.001), ****p < 0.0001)

Relative gene expression of five pro-inflammatory markers (four cytokines (IL-1β, IL-1α, TNFα and IL-8) and one cell surface marker (CCR7) is shown in Fig. 1a. The positive LPS control significantly upregulated all pro-inflammatory markers, except for IL-1α and TNFα on day 5.

The only suture that caused a statistically significant IL-1β upregulation in the THP-1 cells was Perma-Hand® on day 3 with an approximate 60-fold expression increase compared to untreated cells (*p* < 0.05). Other notable IL-1β upregulation against negative control came from cell exposed to Perma-Hand® on day 1, VICRYL® on days 1 and 3, Ti-Cron® on days 3 and 5, and FibreWire® on day 5, but these were not statistically significant. Cells exposed to MONOCRYL®, Ethilon® and Ethibond EXCEL® produced similar levels of IL-1β expression to negative control over all time points.

IL-1α was expressed at the lowest levels of all pro-inflammatory markers. No suture material caused significant increases in IL-1α expression compared to the control cells. Sutures caused varying levels of IL-1α upregulation, generally peaking on day 3. VICRYL®, Ti-Cron® and Perma-Hand® did upregulate IL-1α expression in cells on day 3, however, none of these were statistically significant. Again, cells exposed to MONOCRYL® and Ethilon® produced similar levels of IL-1α expression to negative control over all time points.

TNFα was the pro-inflammatory marker that was upregulated most by all sutures. Increases in expression varied for sutures across the culture period, but all peaked on day 3. Of all sutures, Ti-Cron® caused the greatest increase in TNFα expression, with significant increases of 3340-fold (*p* < 0.0001) and 920-fold (*p* < 0.01) on days 3 and 5, respectively. FiberWire® also caused significant increases, with 2600-fold (*p* < 0.0001) and 1940-fold (*p* < 0.0001) increases in expression seen on days 3 and 5, respectively. Ethibond EXCEL® also significantly increased TNFα upregulation on day 3 with 1990-fold (*p* < 0.0001) expression. Cells exposed to MONOCRYL® and Ethilon® produced similar levels of TNFα expression to negative control over all time points.

The most significant upregulation of IL-8 expression, compared to the negative control, came from exposure to VICRYL® on day 3, with a 50-fold increase in relative gene expression (*p* < 0.0001). The next highest was Ti-Cron® on days 3 and 5 with 45-fold (*p* < 0.0001) and 20-fold (*p* < 0.01) increases, respectively. Perma-Hand® also significantly increased IL-8 expression in THP-1 cells on day 3 with a 38-fold increase (*p* < 0.0001). Other notable increases above baseline came from exposure to VICRYL® and Perma-Hand® on day 1, Ethibond EXCEL® on day 3, and FiberWire® on day 5. However, these did not reach statistical significance. Peak levels of IL-8 expression came mostly on day 3. Once more, cells exposed to MONOCRYL® and Ethilon® produced similar levels of IL-8 expression to negative control over all time points.

Of all sutures, Perma-Hand® caused the greatest increase in CCR7 expression in the THP-1 cells, compared to negative control, with a statistically significant 34-fold increase seen on day 3 (*p* < 0.0001). VICRYL® also caused a significant increase in CCR7 expression on this day with a 15-fold increase (*p* < 0.05). At other time points, exposure to these sutures, along with all others, resulted in expression similar to or reduced from negative control.

#### Anti-inflammatory markers

Relative gene expression of three anti-inflammatory markers (two cytokines (TGFβ1 and IL-1RA) and one cell surface marker (CD163) is shown in Fig. 1b. The positive LPS control produced statistically significant increases in the expression of TGFβ1 on day 1, IL-1RA on days 1 and 3, and CD163 on day 3.

TGFβ1 was expressed at the lowest levels for all anti-inflammatory markers. Ti-Cron® caused the greatest increase in TGFβ1 expression on day 3, with a significant 4.5-fold increase (*p* < 0.001). Exposure to all other sutures caused either similar or reduced levels of TGFβ1 expression in the THP-1 cells compared to negative control, over all days.

All sutures caused a peak in IL-1RA expression levels in the THP-1 cells on day 3. Of all sutures, Perma-Hand® caused the greatest upregulation in IL-1RA expression, with a significant 12.5-fold increase on day 3 (*p* < 0.0001). THP-1 cells exposed to Ti-Cron® produced the next highest, with a statistically significant 10.5-fold increase when compared to the negative control cells on day 3 (*p* < 0.0001). There were also significant increases in IL-1RA expression on day 3 from cells exposed to Ethilon® and Ethibond EXCEL® sutures, with both producing approximately 7.5-fold increase (*p* < 0.05). Exposure to all other sutures on this day, as well as all suture materials on days 1 and 5, produced IL-1RA expression similar to or reduced from negative control.

No THP-1 cells exposed to suture expressed CD163 on day one. Perma-Hand® caused the highest increase in CD163 expression, compared to negative control, on day 3 with a statistically significant 43-fold increase (*p* < 0.0001), compared to negative control. This suture also caused a significant 11-fold increase in CD163 expression on day 5 (*p* < 0.05). Moreover, CD163 was significantly upregulated 14-fold by exposure to VICRYL® on day 3 (*p* < 0.01). Ethilon® and Ti-Cron® had increased CD163 expression on days 3 and 5, as well as FiberWire® on day 5, however, these did not reach statistical significance. MONOCRYL® and Ethibond EXCEL® produced similar levels of CD163 expression to negative control across the culture period.

#### IL-1β/IL-1RA ratio

**Fig. 2:**
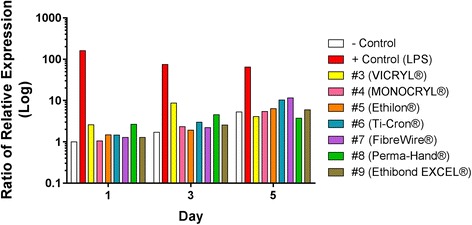
Pro-/Anti-Inflammatory Marker Ratio. A pro-/anti-inflammatory marker ratio was produced for all sutures, by dividing the value for fold-increase in IL-1β gene expression, by the value for fold-increase in IL-1RA gene expression, over all time points

A pro-/anti-inflammatory marker ratio was produced for all sutures by dividing the value for fold-increase in IL-1β gene expression, by the value for fold-increase in IL-1RA gene expression, the inhibitor of IL-1β, over all time points (Fig. 2). This was done to predict the balance of pro- and anti-inflammatory expression elicited in macrophage cells exposed to the suture materials. Compared to negative control, VICRYL® and Perma-Hand® achieve a ratio skewed towards pro-inflammation on days 1 and 3, with values of 2.5 and 8.5, and 2.7 and 4.5, respectively. The Ti-Cron® suture favoured pro-inflammation on days 3 and 5 with ratios of 3 and 10 respectively. FiberWire® produced a propensity for pro-inflammation on day 5, with a ratio of 11.5. MONOCRYL®, Ethilon® and Ethibond EXCEL® produced similar IL-1β:IL-1RA ratios to negative control over all time points.

### Protein secretion analysis

**Fig. 3:**
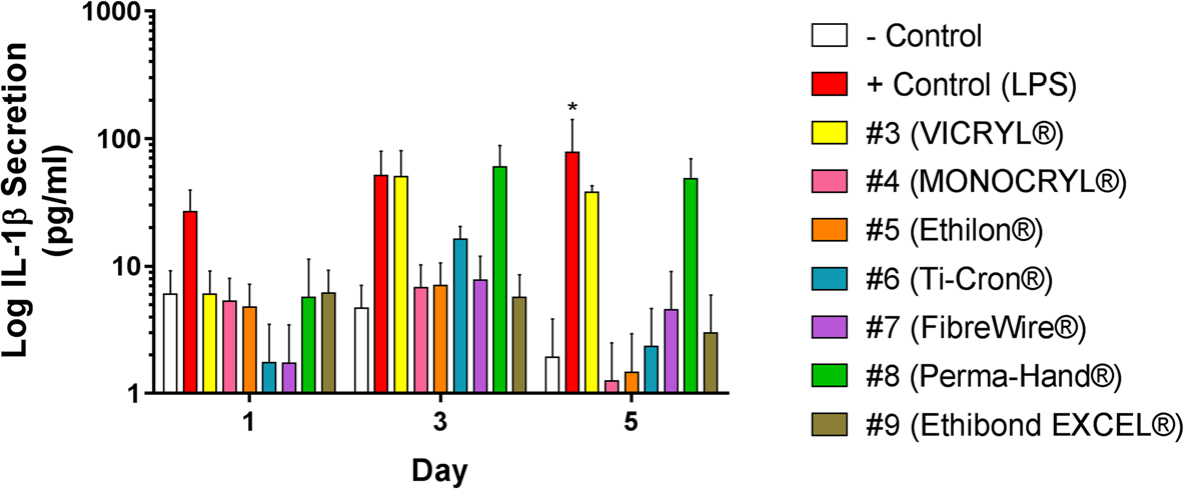
Protein Secretion Results. The concentration of IL-1β protein released by THP-1 cells into media, in response to culture with the suture materials, was measured using ELISA on days 1, 3 and 5. Means from three independent biological repeats is reported ± SEM. Statistical analysis was performed using two-way ANOVA and post-hoc Dunnett’s test (*p < 0.05)

The concentration of IL-1β protein released by THP-1 cells into media, in response to culture with the suture materials, was measured using ELISA on days 1, 3 and 5. Means from three combined independent biological experiments are reported (Fig. 3).

LPS upregulated IL-1β secretion over the entire culture period, however, was only statistically significant on day 5 (*p* < 0.05). IL-1β protein secretion matched the IL-1β gene expression data with peak secretion levels coming on day 3 for all suture materials. Of all suture materials, exposure to Perma-Hand® produced the greatest increase in IL-1β secretion over negative control, with 60 and 48pg/ml secretion on days 3 and 5, respectively. The next highest increase came from THP-1 cells in contact with VICRYL® on days 3 and 5, with 50 and 38pg/ml, respectively. Ti-Cron® also upregulated IL-1β on day 3 with 16pg/ml secretion. However, none of these reached statistical significance. All other sutures had IL-1β secretion levels similar to, or reduced below negative control values across the culture period.

## Discussion

This is the first comprehensive study directly comparing immune response of suture materials, in vitro. The findings from our in vitro study suggest that VICRYL®, Ti-Cron®, FiberWire® and Perma-Hand® suture materials cause upregulation of pro-inflammatory marker genes early in the foreign body reaction, while MONOCRYL®, Ethilon® and Ethibond EXCEL® sutures remain reasonably inert. Pro-/anti-inflammatory ratios convey no balancing from anti-inflammatory marker genes and protein secretion analysis matched gene expression data for IL-1β over the culture period.

The term foreign body reaction describes the body’s inflammatory reaction to foreign objects, such as suture materials. The action of introducing a suture material for wound closure in a living host causes insult, releasing activational cytokines and initiating a chain of inflammation. There are two main macrophage sub-types which play a central and critical role in wound healing at the foreign body site, M1 (classically activated) and M2 (alternatively activated) [16].

The M1 sub-type is characterised by the release of large amounts of pro-inflammatory cytokines, including IL-1β, IL-1α, IL-6, IL-8, IL-12 and TNFα, and by surface marker expression, principally CCR7. The M1 lineage functions in pro-local and systemic inflammation, and if persistent around sites of suture deposition, result in less than optimal wound healing [15, 16]. The M2 sub-type has three independent types, of which the M2c type is the most relevant for suture site repair. Conversely, these macrophages are known to promote wound healing, remodelling and extracellular matrix deposition, by releasing large amounts of anti-inflammatory cytokines such as TGFβ, IL-1RA and IL-10. M2c macrophages can also be characterised by surface marker expression, principally CD163 and cluster of differentiation 206 (CD206) [15, 16].

In this study, relative gene expression of six inflammatory cytokines (IL-1β, IL-1α, TNFα, IL-8, TGFβ1 and IL-RA) and two cell surface markers (CCR7 and CD163) were measured in vitro to assess immune compatibility for seven surgical suture materials. These markers were chosen because they represented a combination of both M1 and M2c markers, allowing assessment of both pro-/anti-inflammatory response to the suture materials. Moreover, their detection has consistently been shown in previous literature assessing immune response to other biomaterials [15, 17–19]. A balance was chosen such that measuring additional markers would be neither economical nor likely to add further information.

Here, we present an in vitro co-incubation model of different suture materials with the human monocyte/macrophage cell line, THP-1. THP-1 cells are homogenous monocytic cells, developed from the peripheral blood of a one-year old infant with acute monocytic leukaemia, that can be activated into mature macrophage cells in vitro [20]. As an immortalized cell line, that can grow and divide indefinitely in vitro, there are certain limitations regarding the conclusions that can be drawn in relation to in vivo monocyte/macrophage response. However, a recent study compared the ability of THP-1 cells to mimic monocytes/macrophages, and determined that, along with the low variability and higher reproducibility of a cell line, under certain conditions, THP-1 cells are capable of resembling primary monocyte/macrophages isolated from healthy patients [21].

Seven suture materials used in general soft tissue approximation were assessed for in vitro immune response. VICRYL® and MONOCRYL® are absorbable sutures which remain inside the body, and are broken down over many months following implantation. VICRYL® is a synthetic copolymer composed of 90% glycolide and 10% L-lactide, while MONOCRYL® is a monofilament synthetic copolymer of glycolide and epsilon-caprolactone [22]. The remaining five suture materials are non-absorbable, meaning they must be removed after enough time has passed to allow sufficient wound healing. Usually, this time period is at least 10 days duration [22]. These sutures were Ethilon®, a monofilament suture composed of the long chain aliphatic polymers Nylon 6 and Nylon 6,6 [22], Ti-Cron®, a suture composed of polyethylene terephthalate with a silicone coating [7], FiberWire®, composed of a braided polyester jacket with a polyethylene core [8], Perma-Hand®, a silk suture composed of an organic fibroin protein derived from the *Bombyx mori* silkworm [23], and finally, Ethibond EXCEL®, a suture composed of braided polyethylene terephthalate, with a polybutilate coat [22].

Our gene expression results suggest that VICRYL®, Ti-Cron®, FiberWire® and Perma-Hand® suture materials cause upregulation of pro-inflammatory markers early in the foreign body reaction, with no balancing from anti-inflammatory markers. In fact, all anti-inflammatory markers measured were expressed at much lower levels compared with their pro-inflammatory counterparts, and this observation was reflected in the increased IL-1β/IL-1RA ratio for VICRYL®, Ti-Cron®, FiberWire® and Perma-Hand®. The protein secretion results matched this gene data for IL-1β expression over the culture period, adding the validity of translational data with transcriptional. Pro-inflammation seemed to peak on day three of culture for most markers assessed, however, several of the pro-inflammatory markers persisted to be high in an absolute sense on day five of culture. Prolonged pro-inflammation, with no subsequent anti-inflammatory peak, is known to produce delayed wound healing responses [24–26]. Such early unbalanced immune reactions can jeopardise success of healing, and this has been shown both with cutaneous wound healing and in models of bony non-union [27–29]. Certainly, persistent pro-inflammation would be expected to hinder wound healing around suture implant sites as well. The three other sutures tested, MONOCRYL®, Ethilon® and Ethibond EXCEL®, remained reasonably inert over the entire culture period.

The IL-1β/IL-1RA ratio used in this study was calculated as a simple measure of whether inflammation was skewed more toward an M1 or M2c type of macrophage response. This ratio supplements the absolute data for pro-/anti-inflammatory cytokine expression. A disadvantage of using this ratio is that absolute changes in individual cytokine expression on either side of the ratio, might not represent biologically equal impact [30, 31]. Using multiple M1 and M2c markers when creating the ratio may reduce some error from this, although measuring protein secretion data may be more reliable.

The authors could see no clear commonality between the four sutures which would suggest a reason for the increased pro-inflammatory responses. Furthermore, we feel it is not possible to rank those sutures in order of response, as each increased different pro-inflammatory cytokines to varying degrees. However, we feel this study does provide useful data towards the options surgeons have, when choosing between various absorbable and non-absorbable suture varieties required in wound closure. Our data would suggest that, if a surgeon requires an absorbable suture, then MONOCRYL® would produce less inflammatory response compared with VICRYL®. Moreover, Ethilon® and Ethibond EXCEL® sutures would produce less inflammation compared with Ti-Cron®, FiberWire® and Perma-Hand®. A reduced inflammatory response would expectantly produce better wound healing and wound closure, with less chance of SRPI and granuloma formation.

In vivo literature is available for some of these sutures, amongst others, with comparable results to our in vitro assessment. Carr et al. (2009) evaluated 8 common sutures (Ethibond EXCEL®, Ti-Cron®, HiFi®, Ultrabraid®, MaxBraid®, Orthocord®, MagnumWire® and FiberWire®) in a rabbit model and graded them up to 120 days post implant, concluding MagnumWire® and Ti-Cron® to stimulate the most intense inflammatory responses [5]. Other studies have been undertaken in rats. Molea et al. (2000) evaluated three bioresorbable monofilament sutures (PDS II®, MONOCRYL® and Biosyn®) up to 6 months post-implantation [32]. Despite all three materials being accepted, PDS II® suture showed an increased histological inflammatory reaction. Other rat studies have evaluated PDS II®, MONOCRYL®, VICRYL®, Chromic Gut® and e-PTFE®. Despite acceptance from the animals, VICRYL® suture showed the highest level of inflammation on histological evaluation [33, 34].

Although in vivo studies have often been deemed the gold standard for assessing immune response, here we have demonstrated in vitro techniques with the sensitivity to distinguish between materials likely to induce a foreign body response, and those unlikely to, and these results largely mirror the previous in vivo studies [5, 32–34]. Given that there is a large global push for a reduction in experimental animal usage [11], we feel that similar in vitro testing is a viable, and ethically friendly option that could be further utilised to evaluate materials used in surgery, such as other suture materials and suture anchors. This would provide surgeons with the information required to make informed surgical decisions, and ultimately improve patient safety.

## Conclusions

Our findings suggest that VICRYL®, Ti-Cron®, FiberWire® and Perma-Hand® suture materials cause the upregulation of pro-inflammatory cytokines indicative of early foreign body reaction. This work has the potential to not only reduce suture-related adverse immune reactions, but the increased knowledge produced from this work will improve informed surgical decision making and ultimately enhance patient safety.

## Abbreviations

ANOVA: Analysis of variance
CCR7: C-C chemokine receptor Type 7
CD163: Cluster of differentiation 163
CD206: Cluster of differentiation 206
ELISA: Enzyme-linked immunosorbent assay
IL-10: Interleukin-10
IL-12: Interleukin-12
IL-1RA: Interleukin-1 receptor antagonist
IL-1α: Interleukin-1 alpha
IL-1β: Interleukin-1 beta
IL-6: Interleukin-6
IL-8: Interleukin-8
LPS: Lipopolysaccharide
M1: Classically activated macrophage sub-type (Pro-inflammatory)
M2: Alternatively activated macrophage sub-type
M2c: Type of alternatively activated macrophages (Anti-inflammatory)
qPCR: Quantitative polymerase chain reaction
RPMI: Roswell park memorial institute
SRPI: Suture-related pseudoinfection
TGFβ: Transforming growth factor beta
TNFα: Tumour necrosis factor alpha
